# Supplementary material for: scVGATAE: A Variational Graph Attentional Autoencoder Model for Clustering Single-Cell RNA-seq Data
Source: Biology (Basel). 2024 Sep 11;13(9):713. doi: 10.3390/biology13090713 (PMC11428844; doi:10.3390/biology13090713)
Supplement: Supplementary file 1 [file biology-13-00713-s001.zip › biology-3163904-supplementary.pdf]

Supplementary Materials — — ‘scVGATAE: a variational graph attentional autoencoder model for clustering single-cell RNA-seq data’

Lijun Liu, Xiaoyang Wu, Jun Yu *, Yuduo Zhang, Kaixing Niu and Anli Yu

School of Science, Dalian Minzu University, Dalian 116600, China

* Correspondence: yshshng@163.com

SupplementaryTables

Supplementary Table S1. ARI scores of scVGATAE and six comparison methods in 9 datasets.

	scVGATAE	scGAC	scGNN2.0	scASGC	scDeepCluster	scVI	Leiden
Biase	1	1	0.609	1	1	0.899	0.878
PBMC6k	0.741	0.65	0.584	0.579	0.619	0.513	0.508
Goolam	0.776	0.544	0.518	0.459	0.543	0.544	0.394
Adipose	0.674	0.686	0.515	0.272	0.254	0.4	0.466
Liver	0.654	0.432	0.457	0.502	0.51	0.565	0.526
Cerebellum	0.796	0.746	0.586	0.621	0.576	0.309	0.667
Heart	0.601	0.409	0.305	0.536	0.476	0.392	0.532
Kolodzisjczyk	0.757	0.537	0.315	0.783	0.575	0.44	0.416
Baron	0.752	0.642	0.343	0.612	0.67	0.62	0.609
Average	0.750	0.627	0.470	0.596	0.580	0.520	0.555

Supplementary Table S2. NMI scores of scVGATAE and six comparison methods in 9 datasets.

	scVGATAE	scGAC	scGNN2.0	scASGC	scDeepCluster	scVI	Leiden
Biase	1	1	0.569	1	1	0.887	0.851
PBMC6k	0.74	0.735	0.671	0.742	0.691	0.695	0.717
Goolam	0.836	0.734712	0.528	0.645	0.707	0.721	0.668
Adipose	0.631	0.593	0.458	0.417	0.391	0.41	0.548
Liver	0.73	0.653	0.578	0.675	0.696	0.673	0.654
Cerebellum	0.829	0.677579	0.637	0.69	0.693	0.616	0.605
Heart	0.694	0.533	0.478	0.6534	0.599	0.509	0.63
Kolodzisjczyk	0.684	0.572	0.532	0.724	0.607	0.677	0.657
Baron	0.794	0.569	0.635	0.718	0.678	0.762	0.657
Average	0.771	0.674	0.565	0.696	0.674	0.661	0.665

Supplementary Table S3.ARI scores of scVGATAE and its four different ablation study subjects.

	scVGATAE	scVGATAE_no_NE	scVGATAE_no_attention	scVGATAE_all_attention	scVGATAE_no_self-adaption-epochs
Biase	1	1	0.956	0.964	0.619
PBMC6k	0.741	0.692	0.654	0.675	0.633
Goolam	0.776	0.705	0.694	0.729	0.748
Adipose	0.499	0.467	0.435	0.474	0.674
Liver	0.654	0.576	0.603	0.598	0.542
Cerebellum	0.796	0.728	0.69	0.643	0.562
Heart	0.601	0.568	0.437	0.541	0.539
Kolodzisjczyk	0.757	0.721	0.688	0.634	0.64
Baron	0.752	0.693	0.697	0.675	0.597

Supplementary Table S4.NMI scores of scVGATAE and its four different ablation study subjects.

	scVGATAE	scVGATAE_no_NE	scVGATAE_no_attention	scVGATAE_all_attention	scVGATAE_no_self-adaption-epochs
Biase	1	1	0.956	0.964	0.722
PBMC6k	0.74	0.706	0.644	0.698	0.654
Goolam	0.836	0.786	0.766	0.719	0.785
Adipose	0.543	0.532	0.522	0.487	0.631
Liver	0.73	0.701	0.703	0.678	0.565
Cerebellum	0.829	0.765	0.754	0.453	0.645
Heart	0.694	0.654	0.581	0.639	0.592
Kolodzisjczyk	0.684	0.67	0.614	0.611	0.675
Baron	0.794	0.738	0.696	0.713	0.729

Supplementary Table S5.Running time of scVGATAE and six comparison methods in 9 datasets.

	scVGATAE	scGAC	scGNN2.0	scASGC	scDeepCluster	scVI	Leiden
Biase	13.95	30.39	804.58	42.15	24	12.83	14.85
Baron	22.35	313.24	2087.54	72.66	196	54.6	20.58
Goolam	46.38	39	1953.24	46.72	54	23.99	16.58
Kolodzisjczyk	62.2	237.2	2364.74	34.92	281	97.84	26.67
Heart	150.68	308.44	1983.07	39.41	265	69.42	23.65
Adipose	129.47	735	3477.93	39.9	268	68.1	18.53
Liver	224.48	470.48	5189.96	59.77	322	75.89	26.97
PBMC6k	250.13	4861.36	7213.78	275.74	1287	235.74	32.25
Cerebellum	530.18	5322.94	8367.45	568.69	2389	296.38	106.45
